# Delayed Adverse Reactions Following Whole-Blood and Plateletpheresis Donation: A Prospective Observational Study From a Tertiary-Care Blood Center

**DOI:** 10.7759/cureus.115261

**Published:** 2026-08-26

**Authors:** Sunil Kumar, Tulika Chandra, Archana Solanki, Ashutosh Singh, Neelam Kumari

**Affiliations:** 1 Transfusion Medicine, King George's Medical University, Lucknow, IND; 2 Physical Medicine and Rehabilitation, King George's Medical University, Lucknow, IND

**Keywords:** blood donation, blood donor complications, delayed adverse reactions, donor hemovigilance, donor safety, plateletpheresis, telephonic follow-up, whole-blood donors

## Abstract

Background and aim: Delayed adverse reactions occurring after donors leave the blood center may be underrecognized by routine on-site hemovigilance. Comparative data on delayed reactions following whole-blood donation and plateletpheresis within the same institutional setting remain limited. This study aimed to determine the frequency and pattern of delayed adverse reactions and evaluate their associations with donor age, sex, donation status, and donation type.

Methods: This prospective observational study included 17,923 donors (17,633 whole-blood and 290 plateletpheresis) at a single tertiary-care blood center from June 2020 to May 2021. Four-week telephone follow-up was completed by 16,292/17,923 (90.9%) donors using a structured interview schedule. Reaction-specific proportions were calculated using the enrolled donation-group denominators. Associations were assessed using Pearson's chi-square or exact tests, as appropriate; odds ratios (ORs) were crude and unadjusted.

Results: Whole-blood donors most frequently reported fatigue (587/17,633 {3.33%}), bruising (390/17,633 {2.21%}), painful arm (283/17,633 {1.61%}), and hematoma (102/17,633 {0.58%}). Among plateletpheresis donors, hematoma (11/290 {3.79%}) and bruising (9/290 {3.10%}) were most frequent. Plateletpheresis was associated with higher unadjusted odds of hematoma (OR: 6.77, 95% CI: 3.57-12.84; p<0.001), allergy (OR: 14.12, 95% CI: 4.03-49.47; p=0.002), delayed bleeding (OR: 17.37, 95% CI: 3.59-84.09; p=0.009), and nerve irritation (OR: 13.58, 95% CI: 2.91-63.35; p=0.013). Among whole-blood donors, bruising, fatigue, and painful arm were more frequent among females (all p<0.001). Plateletpheresis subgroup findings were considered exploratory because of small subgroup numbers.

Conclusions: Delayed-reaction profiles differed across donation modalities, with local venous-access complications more prominent after plateletpheresis. These unadjusted single-center findings require cautious interpretation and multicenter validation.

## Introduction

Blood donation is essential to modern healthcare and depends on maintaining a safe and willing donor population. Although donation is generally well tolerated, adverse reactions may occur before venipuncture, during blood collection, during the immediate post-donation recovery period, or after the donor has left the blood center. Such reactions are usually mild but remain clinically relevant because they may affect donor well-being, confidence, and future willingness to donate [1,2]. The present study specifically evaluated delayed adverse reactions occurring after donors had left the donation center; pre-donation reactions and events occurring before completion of donation were outside the scope of this analysis.

Donor hemovigilance provides a structured framework for identification, documentation, analysis, and prevention of adverse events associated with blood donation. In India, the National Blood Donor Vigilance Program was established within the Hemovigilance Program of India to strengthen standardized reporting and donor safety [1,2]. International consensus definitions have similarly improved the classification of donation-related complications and facilitated comparison across blood services [3]. However, routine surveillance more readily captures reactions occurring during donation or within the immediate observation period, whereas symptoms developing after donors return home may remain underreported. Delayed adverse reactions can include fatigue or generalized weakness, bruising, hematoma, painful arm, delayed vasovagal symptoms, bleeding, allergic manifestations, and nerve-related symptoms [4-7]. Previous studies using structured post-donation contact have shown that delayed symptoms may be detected more frequently than through routine on-site surveillance alone [4,7]. Donor characteristics such as age, sex, estimated blood volume, and donation experience may also influence susceptibility to adverse reactions [6-11].

Whole-blood donation and plateletpheresis differ substantially in their procedures and physiological effects. Whole-blood donation involves removal of a fixed volume of blood through a single venipuncture, whereas plateletpheresis requires prolonged venous access, extracorporeal circulation, component separation and return, and citrate anticoagulation. Consequently, the pattern of donor reactions may differ between the two donation modalities. Existing studies have often evaluated whole-blood and plateletpheresis donors independently, and much of the available evidence has focused on immediate or on-site adverse reactions rather than delayed off-site events [5,11].

Accordingly, the primary objective of this study was to determine the frequency and pattern of delayed adverse reactions following blood donation. The secondary objectives were to compare the frequency and pattern of delayed adverse reactions between whole-blood and plateletpheresis donors and to evaluate their associations with donor age, sex, and donation status. By evaluating both donation modalities within a single tertiary-care blood center using structured four-week telephonic follow-up, this study aimed to address the limited comparative evidence on delayed donor reactions in a uniform institutional setting.

## Materials and methods

Study design, setting, and reporting

This prospective observational study was conducted at the Department of Transfusion Medicine, King George's Medical University (KGMU), Lucknow, India, over a one-year period from June 1, 2020, to May 31, 2021. All donations included in this study were performed at this single tertiary-care blood center; data were not pooled from other donation centers. All eligible consenting donors presenting during the study period were considered for inclusion, without selection of a sampling subset. Both male and female donors were eligible provided that the applicable national donor-selection criteria were fulfilled. Written informed consent was obtained from all participants before enrollment. The reporting of the study follows the Strengthening the Reporting of Observational Studies in Epidemiology (STROBE) recommendations, and the completed STROBE checklist is provided as appendix 1.

Sample size

The minimum sample size was calculated using an expected prevalence of delayed adverse reactions of 10.3% among whole-blood donors, based on the study by Tiwari et al. [12]. A two-sided significance level of 5%, 80% power, an allowable error of 20% of the expected prevalence, and an additional 10% allowance for non-response or data loss were considered, resulting in a minimum required sample size of 920 donors. As all eligible consenting donors presenting during the predefined one-year study period were included, 17,923 donors were ultimately enrolled, substantially exceeding the minimum calculated sample size.

Study population and donor eligibility

Healthy donors fulfilling the donor-selection requirements of the Directorate General of Health Services (DGHS), Ministry of Health and Family Welfare, Government of India, and the applicable Drugs and Cosmetics Rules were eligible for participation. Whole-blood donors were required to fulfill the applicable criteria relating to age, body weight, hemoglobin concentration, vital signs, general medical fitness, medication use, and temporary or permanent deferral. Plateletpheresis donors were required to be 18-60 years of age, weigh ≥50 kg, have hemoglobin ≥12.5 g/dL and platelet count ≥150×10⁹/L, satisfy routine medical-fitness and transfusion-transmitted infection screening requirements, have adequate venous access, and meet the applicable medication and inter-donation interval requirements. First-time donor status was not, by itself, an exclusion criterion for plateletpheresis when all other eligibility requirements were fulfilled. These criteria are consistent with the eligibility information in the current revision. The principal eligibility criteria are summarized in Table 1. Detailed regulatory temporary and permanent deferral criteria are provided in appendix 2 to improve readability.

**Table 1:** Key eligibility criteria for whole-blood and plateletpheresis donors. NSAID: nonsteroidal anti-inflammatory drug

Donation type	Criterion	Inclusion criterion	Exclusion/deferral criterion
Whole blood	Age	18-65 years; first-time donors up to 60 years and repeat donors up to 65 years	Age <18 years or above the applicable upper age limit
	Body weight	≥50 kg	<50 kg
	Hemoglobin	≥12.5 g/dL	<12.5 g/dL
	Pulse	60-100/min and regular	Outside the acceptable range or irregular
	Temperature	Afebrile	Febrile
	Blood pressure	Systolic 100-140 mmHg and diastolic 60-90 mmHg	Outside the acceptable range
	General medical fitness	Medically fit for blood donation following history and physical examination	Any clinical finding or medical condition incompatible with donor eligibility
	Temporary/permanent deferral	No applicable temporary or permanent deferral condition at the time of donation	Any applicable illness, medication, procedure, exposure, or permanent medical condition requiring deferral under the donor-selection criteria
Plateletpheresis	Age	18-60 years	<18 or >60 years
	Body weight/medical fitness	≥50 kg and medically fit	<50 kg or medically unfit
	Hemoglobin	≥12.5 g/dL	<12.5 g/dL
	Platelet count	≥150×10⁹/L	<150×10⁹/L
	Transfusion-transmitted infection screening	Non-reactive for mandatory transfusion-transmitted infections	Reactive for any mandatory transfusion-transmitted infection
	NSAID/aspirin exposure	No NSAID/aspirin use during the preceding 72 h	NSAID/aspirin use during the preceding 72 h
	Venous access	Suitable antecubital venous access	Unsuitable venous access
	Interval between plateletpheresis procedures	≥48 h; not more than two procedures/week or 24 procedures/year	Interval <48 h or frequency exceeding two procedures/week or 24 procedures/year
	Previous plateletpheresis reaction	No history of adverse donor reaction during previous plateletpheresis donation	History of adverse donor reaction during previous plateletpheresis donation
	Other deferral conditions	Fulfillment of applicable medication and donor-deferral criteria	Any applicable medication-related or other donor-deferral criterion

Blood donation procedures

Whole-blood donation was performed according to standard blood-center procedures using sterile, single-use blood-collection systems. Approximately 350 mL of whole blood was collected from an antecubital vein. Following donation, donors remained under observation for approximately 10 min, received refreshments, and were provided standard post-donation instructions before leaving the blood center. Plateletpheresis was performed using the COM.TEC cell separator (Bad Homburg, Germany: Fresenius Kabi AG) with sterile disposable apheresis kits and acid citrate dextrose anticoagulant. Donor demographic, clinical, and hematological parameters were entered into the apheresis system before initiation of the procedure. Platelet yield and procedure-related adverse reactions were documented following completion of plateletpheresis.

Scope and classification of adverse reactions

Donation-related adverse reactions may occur before venipuncture, during blood collection, during the immediate post-donation period, or after the donor has left the blood center. The present study specifically evaluated delayed/off-site adverse reactions following completed donations. Pre-donation reactions, including events occurring before venipuncture or that prevented completion of donation, were outside the scope of the present analysis. For this study, reactions occurring during donation or while the donor remained at the blood center were considered early/on-site reactions, whereas symptoms occurring after the donor had left the donation facility were considered delayed/off-site reactions. The delayed reaction categories assessed during follow-up included bruising, hematoma, painful arm, fatigue/weakness, vasovagal reaction, allergy, delayed bleeding, and nerve irritation.

Operational assessment of delayed reactions

Delayed reactions were identified through donor self-report during the structured telephone interview rather than by direct physical examination. To facilitate differentiation of local reactions, bruising was described as bluish discoloration around the venipuncture site without swelling, whereas hematoma was characterized by discoloration accompanied by swelling. Painful arm referred to post-donation pain or discomfort involving the phlebotomy arm. Allergic manifestations included reported redness and/or itching, while delayed bleeding referred to bleeding from the venipuncture site after the donor had left the blood center. Fatigue was assessed as self-reported fatigue or generalized weakness. Nerve irritation was recorded on the basis of post-donation sensory symptoms such as numbness or tingling reported after leaving the blood center. In plateletpheresis donors, citrate-associated manifestations occurring during the procedure, including tingling, burning sensations, muscle cramps, tetany, or related neuromuscular symptoms, were considered acute procedural reactions and were not included as delayed off-site reactions. The temporal relationship to the procedure was therefore used to distinguish acute citrate-associated paresthesia from sensory symptoms subsequently reported after leaving the blood center.

Post-donation telephone follow-up

All enrolled donors were scheduled for telephonic follow-up four weeks after donation using a structured interview schedule. The four-week follow-up interval was predefined in the study methodology and was based on the post-donation telephonic follow-up approach described by Tiwari et al. [12]. During the interview, donors were asked about their general well-being and whether they had experienced discomfort during or after donation, followed by questions addressing the predefined delayed reaction categories, including bruising, hematoma, painful arm, numbness or tingling, fatigue/weakness, allergic symptoms, delayed bleeding, and other relevant post-donation complaints. Donors reporting persistent or concerning symptoms were advised to attend the blood center for assessment. The interview schedule was used as a structured symptom-assessment instrument; however, a separate formal psychometric validation was not performed. Details regarding formal interviewer training/calibration, the professional category of individual callers, and a prespecified number or timing of repeat call attempts are not available in the retained study records and therefore have not been retrospectively inferred. The interview schedule used for post-donation assessment is provided in appendix 3. The four-week interval may have increased susceptibility to recall error, particularly for mild or non-specific symptoms such as fatigue. This potential source of measurement error is acknowledged in the study limitations.

Follow-up completeness and missing follow-up

Of the 17,923 enrolled donors, 16,292 (90.9%) completed telephonic follow-up, while 1,631 (9.1%) did not complete follow-up. The available aggregate study records do not retain the distribution of these 1,631 non-respondents separately for whole-blood and plateletpheresis donors. Consequently, donation-type-specific response rates and comparisons between respondents and non-respondents could not be reconstructed. No assumptions or imputations were made regarding reactions among donors without completed follow-up. Potential non-response bias arising from incomplete follow-up is acknowledged as a study limitation. The available records support the overall follow-up numbers but not donation-type-specific non-response.

Handling of reaction categories

Delayed adverse reactions were classified according to the predefined reaction categories and analyzed separately for whole-blood and plateletpheresis donors. For each reaction category, results are presented as frequency and percentage (n {%}), using the corresponding donation-group denominator. Comparisons between donation types and subgroup analyses according to donor characteristics were performed separately for each reaction category.

Severity grading

The available study records classify delayed reactions according to reaction type but do not contain standardized International Society of Blood Transfusion (ISBT)/International Haemovigilance Network (IHN) severity grades or sufficient participant-level clinical information for reliable retrospective severity classification. Consequently, post hoc severity grading was not undertaken, as doing so would require assumptions not supported by the available data. Lack of standardized severity grading is acknowledged as a limitation.

Outcome measures

The primary outcome was the frequency and pattern of reported delayed/off-site adverse reaction categories following donation. The secondary outcomes were the comparison of the frequency and pattern of delayed adverse reactions between whole-blood and plateletpheresis donation, and the assessment of the associations of delayed reaction categories with donor age, sex, and donation status (first-time vs. repeat donor).

Statistical analysis

Data were entered into Microsoft Excel (Redmond, WA: Microsoft Corp.) and analyzed using SPSS version 23.0 (Armonk, NY: IBM Corp.) statistics for Windows. Categorical variables were summarized as frequencies and percentages. Baseline characteristics were compared between whole-blood and plateletpheresis donors using Pearson's chi-square test. The chi-square statistic (χ²), degrees of freedom (df), and effect size were reported for these comparisons. The Phi coefficient (φ) was used as the effect-size measure for 2×2 contingency tables, whereas Cramér’s V was used for contingency tables larger than 2×2. Individual delayed adverse reactions were compared between whole-blood and plateletpheresis donors using Pearson's chi-square test when the assumptions of the test were satisfied. Fisher’s exact test was used for comparisons involving sparse cells or small expected frequencies. Crude odds ratios (ORs) with 95% confidence intervals (CIs) were calculated to quantify the unadjusted association between donation type and individual delayed adverse reactions. Among whole-blood donors, associations of delayed adverse reactions with age group, sex, and donation status were assessed using Pearson's chi-square test when appropriate and exact tests for sparse contingency tables. Effect sizes were reported as Phi coefficients or Cramér’s V, as applicable. Among plateletpheresis donors, subgroup comparisons according to age group, sex, and donation status were performed using Fisher’s exact test for 2×2 contingency tables and the Fisher-Freeman-Halton exact test for contingency tables with more than two categories. Owing to the small numbers within several plateletpheresis subgroups, these analyses were considered exploratory. Telephone follow-up was completed for 16,292/17,923 (90.9%) enrolled donors. Reaction-specific frequencies and percentages were calculated using the enrolled donation-group denominators of 17,633 whole-blood donors and 290 plateletpheresis donors. No imputation was performed for donors without completed telephone follow-up. All statistical tests were two-tailed, and a p<0.05 was considered statistically significant.

## Results

Baseline characteristics of the study population

A total of 17,923 donors were included, comprising 17,633 (98.4%) whole-blood donors and 290 (1.6%) plateletpheresis donors. The age distribution differed significantly according to donation type (χ²{3}=59.77, p<0.001, Cramér's V=0.058). Among whole-blood donors, 9,408 (53.4%) were aged 18-29 years compared with 97 (33.4%) plateletpheresis donors. Sex distribution also differed between the groups (χ²{1}=25.48, p<0.001, φ=0.038); males accounted for 17,314 (98.2%) whole-blood donors and 273 (94.1%) plateletpheresis donors. Donation status differed significantly by donation type (χ²{1}=85.09, p<0.001, φ=0.069), with first-time donors comprising 12,646 (71.7%) whole-blood donors and 279 (96.2%) plateletpheresis donors. Although statistically significant, the corresponding effect sizes were small (Table 2).

**Table 2:** Baseline characteristics of study participants according to donation type. ^†^Cramér's V. ^‡^Phi coefficient (φ). Percentages were calculated using the total number of donors within each study group. Comparisons between whole-blood and plateletpheresis donors were performed using Pearson's chi-square test. Effect sizes are reported as Cramér's V for the age-group comparison (4x2 contingency table) and the phi coefficient (φ) for 2×2 comparisons.

Characteristics	Overall (N=17,923), n (%)	Whole-blood donors (N=17,633), n (%)	Plateletpheresis donors (N=290), n (%)	χ²	df	Effect size	p-Value
Age group (years)							
18-29	9,505 (53.0)	9,408 (53.4)	97 (33.4)	59.77	3	0.058^†^	<0.001
30-39	5,972 (33.3)	5,855 (33.2)	117 (40.3)				
40-49	2,038 (11.4)	1,975 (11.2)	63 (21.7)				
≥50	408 (2.3)	395 (2.2)	13 (4.5)				
Sex							
Male	17,587 (98.1)	17,314 (98.2)	273 (94.1)	25.48	1	0.038^‡^	<0.001
Female	336 (1.9)	319 (1.8)	17 (5.9)				
Donation status							
First-time donor	12,925 (72.1)	12,646 (71.7)	279 (96.2)	85.09	1	0.069^‡^	<0.001
Repeat donor	4,998 (27.9)	4,987 (28.3)	11 (3.8)				

Comparison of delayed adverse reactions between whole-blood and plateletpheresis donors

The pattern of reported delayed adverse reactions differed between the two donation modalities (Table 3). Compared with whole-blood donors, plateletpheresis donors had significantly higher odds of hematoma (11/290 {3.79%} vs. 102/17,633 {0.58%}; crude OR: 6.77, 95% CI: 3.57-12.84; p<0.001), allergy (3/290 {1.03%} vs. 13/17,633 {0.07%}; crude OR: 14.12, 95% CI: 4.03-49.47; p=0.002), delayed bleeding (2/290 {0.69%} vs. 7/17,633 {0.04%}; crude OR: 17.37, 95% CI: 3.59-84.09; p=0.009), and nerve irritation (2/290 {0.69%} vs. 9/17,633 {0.05%}; crude OR: 13.58, 95% CI: 2.91-63.35; p=0.013}. In contrast, differences between plateletpheresis and whole-blood donors were not statistically significant for fatigue (5/290 {1.72%} vs. 587/17,633 {3.33%}; crude OR: 0.51, 95% CI: 0.21-1.24; p=0.129), bruising (9/290 {3.10%} vs. 390/17,633 {2.21%}; crude OR: 1.42, 95% CI: 0.72-2.79; p=0.307), painful arm (4/290 {1.38%} vs. 283/17,633 {1.61%}; crude OR: 0.86, 95% CI: 0.32-2.33; p=1.0), or vasovagal reactions (2/290 {0.69%} vs. 53/17,633 {0.30%}; crude OR: 2.28, 95% CI: 0.55-9.47; p=0.223).

**Table 3:** Comparison of reported delayed adverse reactions between whole-blood and plateletpheresis donors. ^†^Odds ratios are crude (unadjusted) and are expressed for plateletpheresis relative to whole-blood donation. ^‡^The phi coefficient (φ) is reported as the effect-size measure for Pearson's chi-square comparisons. Percentages were calculated using the enrolled donation-group denominators. Fisher's exact test was used when expected cell frequencies were <5; χ² statistics, degrees of freedom, and phi coefficients are therefore not applicable to these comparisons.

Delayed adverse reaction	Whole-blood donors (N=17,633), n (%)	Plateletpheresis donors (N=290), n (%)	Crude OR (95% CI)^†^	Test statistic/test	df	Effect size^‡^	p-Value
Fatigue	587 (3.33)	5 (1.72)	0.51 (0.21-1.24)	χ²=2.30	1	φ=0.011	0.129
Bruising	390 (2.21)	9 (3.10)	1.42 (0.72-2.79)	χ²=1.04	1	φ=0.008	0.307
Painful arm	283 (1.61)	4 (1.38)	0.86 (0.32-2.33)	Fisher's exact	-	-	1.0
Hematoma	102 (0.58)	11 (3.79)	6.77 (3.57-12.84)	Fisher's exact	-	-	<0.001
Vasovagal reaction	53 (0.30)	2 (0.69)	2.28 (0.55-9.47)	Fisher's exact	-	-	0.223
Allergy	13 (0.07)	3 (1.03)	14.12 (4.03-49.47)	Fisher's exact	-	-	0.002
Delayed bleeding	7 (0.04)	2 (0.69)	17.37 (3.59-84.09)	Fisher's exact	-	-	0.009
Nerve irritation	9 (0.05)	2 (0.69)	13.58 (2.91-63.35)	Fisher's exact	-	-	0.013

Distribution of reported delayed adverse reactions according to donor characteristics among whole-blood donors

Among whole-blood donors, several delayed-reaction categories varied according to donor characteristics (Table 4). Fatigue differed significantly across age groups (χ²{3}=68.65, p<0.001, Cramér's V=0.062), with the highest frequency among donors aged 40-49 years (126/1,975 {6.38%}). Painful arm also differed by age (χ²{3}=35.13, p<0.001, Cramér's V=0.045) and was most frequent among donors aged 18-29 years (198/9,408 {2.10%}). Hematoma was also associated with age (exact p<0.001), with the highest frequency among donors aged 18-29 years (92/9,408 {0.98%}). Female whole-blood donors had substantially higher frequencies of bruising (88/319 {27.59%} vs. 302/17,314 {1.74%}; χ²{1}=967.14, p<0.001, φ=0.234), fatigue (111/319 {34.80%} vs. 476/17,314 {2.75%}; χ²{1}=999.61, p<0.001, φ=0.238), and painful arm (63/319 {19.75%} vs. 220/17,314 {1.27%}; χ²{1}=677.27, p<0.001, φ=0.196) than male donors. Repeat donors had higher frequencies of bruising (129/4,987 {2.59%} vs. 261/12,646 {2.06%}; χ²{1}=4.52, p=0.033, φ=0.016) and fatigue (201/4,987 {4.03%} vs. 386/12,646 {3.05%}; χ²{1}=10.63, p=0.001, φ=0.025) than first-time donors. Conversely, vasovagal reactions were more frequent among first-time donors (48/12,646 {0.38%}) than repeat donors (5/4,987 {0.10%}; χ²{1}=9.31, p=0.002, φ=0.023). Other examined associations were not statistically significant.

**Table 4:** Distribution of reported delayed adverse reactions according to donor characteristics among whole-blood donors. ^§^Fisher-Freeman-Halton exact test was used for sparse multi-category contingency tables. Percentages were calculated using the corresponding subgroup denominator. Pearson's chi-square test was used when its assumptions were satisfied. For sparse contingency tables, Fisher's exact test (2×2) or an exact test for larger contingency tables was used as appropriate. Effect sizes are reported as Cramér's V (V) for age-group comparisons and the phi coefficient (φ) for 2×2 Pearson's chi-square comparisons. Effect sizes are not reported for exact-test comparisons. All tests were two-tailed.

Delayed adverse reaction	Age group (years), n/N (%)	Test statistics; p-Value	Effect size	Sex, n/N (%)	Test statistics; p-Value	Effect size	Donation status, n/N (%)	Test statistic; p-Value	Effect size
Bruising	18-29: 193/9,408 (2.05), 30-39: 133/5,855 (2.27), 40-49: 51/1,975 (2.58), ≥50: 13/395 (3.29)	χ²(3)=4.60; p=0.204	0.016	Male: 302/17,314 (1.74); female: 88/319 (27.59)	χ²(1)=967.14; p<0.001	0.234	First-time: 261/12,646 (2.06); repeat: 129/4,987 (2.59)	χ²(1)=4.52; p=0.033	0.016
Fatigue	18-29: 262/9,408 (2.79), 30-39: 181/5,855 (3.09), 40-49: 126/1,975 (6.38), ≥50: 18/395 (4.56)	χ²(3)=68.65; p<0.001	0.062	Male: 476/17,314 (2.75); female: 111/319 (34.80)	χ²(1)=999.61; p<0.001	0.238	First-time: 386/12,646 (3.05); repeat: 201/4,987 (4.03)	χ²(1)=10.63; p=0.001	0.025
Painful arm	18-29: 198/9,408 (2.10), 30-39: 69/5,855 (1.18), 40-49: 15/1,975 (0.76), ≥50: 1/395 (0.25)	χ²(3)=35.13; p<0.001	0.045	Male: 220/17,314 (1.27); female: 63/319 (19.75)	χ²(1)=677.27; p<0.001	0.196	First-time: 189/12,646 (1.49); repeat: 94/4,987 (1.88)	χ²(1)=3.45; p=0.063	0.014
Hematoma	18-29: 92/9,408 (0.98), 30-39: 8/5,855 (0.14), 40-49: 2/1,975 (0.10), ≥50: 0/395 (0)	Exact test^§^; p<0.001	-	Male: 98/17,314 (0.57); female: 4/319 (1.25)	Fisher's exact; p=0.114	-	First-time: 66/12,646 (0.52); repeat: 36/4,987 (0.72)	χ²(1)=2.49; p=0.115	0.012
Delayed bleeding	18-29: 7/9,408 (0.07), 30-39: 0/5,855 (0), 40-49: 0/1,975 (0), ≥50: 0/395 (0)	Exact test^§^; p=0.190	-	Male: 7/17,314 (0.04); female: 0/319 (0)	Fisher's exact; p=1.0	-	First-time: 5/12,646 (0.04); repeat: 2/4,987 (0.04)	Fisher's exact; p=1.0	-
Allergy	18-29: 7/9,408 (0.07), 30-39: 4/5,855 (0.07), 40-49: 2/1,975 (0.10), ≥50: 0/395 (0)	Exact test^§^; p=0.972	-	Male: 12/17,314 (0.07); female: 1/319 (0.31)	Fisher's exact; p=0.211	-	First-time: 8/12,646 (0.06); repeat: 5/4,987 (0.10)	Fisher's exact; p=0.537	-
Vasovagal reaction	18-29: 39/9,408 (0.41), 30-39: 9/5,855 (0.15), 40-49: 4/1,975 (0.20), ≥50: 1/395 (0.25)	Exact test^§^; p=0.061	-	Male: 51/17,314 (0.29); female: 2/319 (0.63)	Fisher's exact; p=0.249	-	First-time: 48/12,646 (0.38); repeat: 5/4,987 (0.10)	χ²(1)=9.31; p=0.002	0.023

Distribution of reported delayed adverse reactions according to donor characteristics among plateletpheresis donors

Because of sparse event counts, plateletpheresis subgroup comparisons were evaluated using exact tests and should be considered exploratory (Table 5). Bruising was more frequent among female donors (3/17 {17.65%}) than male donors (6/273 {2.20%}; Fisher's exact p=0.011) and among repeat donors (2/11 {18.18%}) than first-time donors (7/279 {2.51%}; Fisher's exact p=0.041). Fatigue was also more frequent among female donors (3/17 {17.65%}) than male donors (2/273 {0.73%}; Fisher's exact p=0.002). No other age-, sex-, or donation-status associations reached statistical significance on exact testing. In particular, hematoma did not demonstrate statistically significant associations with age (p=0.082), sex (p=0.130), or donation status (p=0.060). These subgroup findings should be interpreted cautiously because the plateletpheresis cohort included only 17 females and 11 repeat donors.

**Table 5:** Distribution of reported delayed adverse reactions according to donor characteristics among plateletpheresis donors. Because of sparse cell frequencies and very small numbers of events in several plateletpheresis subgroups, Fisher's exact test was used for 2×2 comparisons and the Fisher-Freeman-Halton exact test for age-group comparisons. Effect sizes based on Pearson's chi-square statistic are not reported because exact testing was used. All tests were two-tailed. Subgroup analyses should be considered exploratory because only 17 female and 11 repeat plateletpheresis donors were included.

Delayed adverse reaction	Age group (years), n/N (%)	Statistical test	Sex, n/N (%)	Statistical test	Donation status, n/N (%)	Statistical test
Hematoma	18-29: 8/97 (8.25), 30-39: 2/117 (1.71), 40-49: 1/63 (1.59), 50-59: 0/13 (0)	Fisher-Freeman-Halton exact test; p=0.082	Male: 9/273 (3.30), female: 2/17 (11.76)	Fisher's exact test; p=0.130	First-time: 9/279 (3.23), repeat: 2/11 (18.18)	Fisher's exact test; p=0.060
Bruising	18-29: 7/97 (7.22), 30-39: 1/117 (0.85), 40-49: 1/63 (1.59), 50-59: 0/13 (0)	Fisher-Freeman-Halton exact test; p=0.068	Male: 6/273 (2.20), female: 3/17 (17.65)	Fisher's exact test; p=0.011	First-time: 7/279 (2.51), repeat: 2/11 (18.18)	Fisher's exact test; p=0.041
Fatigue	18-29: 4/97 (4.12), 30-39: 1/117 (0.85), 40-49: 0/63 (0), 50-59: 0/13 (0)	Fisher-Freeman-Halton exact test; p=0.227	Male: 2/273 (0.73), female: 3/17 (17.65)	Fisher's exact test; p=0.002	First-time: 4/279 (1.43), repeat: 1/11 (9.09)	Fisher's exact test; p=0.177
Painful arm	18-29: 3/97 (3.09), 30-39: 1/117 (0.85), 40-49: 0/63 (0), 50-59: 0/13 (0)	Fisher-Freeman-Halton exact test; p=0.464	Male: 3/273 (1.10), female: 1/17 (5.88)	Fisher's exact test; p=0.216	First-time: 4/279 (1.43), repeat: 0/11 (0)	Fisher's exact test; p=1.0
Delayed bleeding	18-29: 1/97 (1.03), 30-39: 1/117 (0.85), 40-49: 0/63 (0), 50-59: 0/13 (0)	Fisher-Freeman-Halton exact test; p=1.0	Male: 2/273 (0.73), female: 0/17 (0)	Fisher's exact test; p=1.0	First-time: 2/279 (0.72), repeat: 0/11 (0)	Fisher's exact test; p=1.0
Allergy	18-29: 1/97 (1.03), 30-39: 0/117 (0), 40-49: 2/63 (3.17), 50-59: 0/13 (0)	Fisher-Freeman-Halton exact test; p=0.223	Male: 3/273 (1.10), female: 0/17 (0)	Fisher's exact test; p=1.0	First-time: 3/279 (1.08), repeat: 0/11 (0)	Fisher's exact test; p=1.0
Nerve irritation	18-29: 0/97 (0), 30-39: 2/117 (1.71), 40-49: 0/63 (0), 50-59: 0/13 (0)	Fisher-Freeman-Halton exact test; p=0.553	Male: 2/273 (0.73), female: 0/17 (0)	Fisher's exact test; p=1.0	First-time: 2/279 (0.72), repeat: 0/11 (0)	Fisher's exact test; p=1.0
Vasovagal reaction	18-29: 2/97 (2.06), 30-39: 0/117 (0), 40-49: 0/63 (0), 50-59: 0/13 (0)	Fisher-Freeman-Halton exact test; p=0.246	Male: 2/273 (0.73), female: 0/17 (0)	Fisher's exact test; p=1.0	First-time: 2/279 (0.72), repeat: 0/11 (0)	Fisher's exact test; p=1.0

## Discussion

The present prospective observational study characterizes the pattern of delayed adverse reactions following whole-blood donation and plateletpheresis at a single tertiary-care blood center. Among whole-blood donors, fatigue (587/17,633 {3.33%}), bruising (390/17,633 {2.21%}), painful arm (283/17,633 {1.61%}), and hematoma (102/17,633 {0.58%}) were the most frequently reported delayed reactions. Among plateletpheresis donors, hematoma (11/290 {3.79%}) and bruising (9/290 {3.10%}) were the most frequent. Compared with whole-blood donation, plateletpheresis was associated with higher unadjusted odds of hematoma, allergy, delayed bleeding, and nerve irritation. These relative differences should be considered alongside their absolute frequencies as follows: although some odds ratios were large, allergy (3/290 {1.03%}), delayed bleeding (2/290 {0.69%}), and nerve irritation (2/290 {0.69%}) remained uncommon. Thus, the clinical relevance of these findings depends not only on statistical significance but also on absolute frequency, clinical consequences, and the potential effect of such reactions on the donor experience.

Structured post-donation follow-up provides an opportunity to identify symptoms that develop after donors have left the blood center and therefore may not be captured during routine immediate observation. Newman and Roth reported that structured interviews conducted after whole-blood donation identified delayed symptoms including fatigue, bruising, and arm discomfort [13]. Orru et al. similarly demonstrated that donor-reported local and systemic symptoms may be incompletely represented in conventional reporting systems [14]. Indian evidence from Gupta and Bajpai also demonstrated the value of prospective follow-up for identifying delayed complaints, particularly generalized weakness and local arm symptoms [15]. In the present study, telephone follow-up was completed for 16,292/17,923 (90.9%) of enrolled donors, enabling systematic assessment of symptoms reported after donors left the blood center. Differences in reported reaction frequencies across studies may reflect variations in donor characteristics, follow-up intervals, symptom definitions, and surveillance methods.

Among whole-blood donors, fatigue was the most frequently reported delayed symptom (587/17,633 {3.33%}), followed by bruising (390/17,633 {2.21%}) and painful arm (283/17,633 {1.61%}). Previous studies have likewise identified fatigue and arm-related complaints among delayed symptoms following whole-blood donation [13]. Fatigue is, however, non-specific, and its attribution to donation should be interpreted cautiously, particularly given the four-week follow-up interval used in the present study. In contrast, bruising, painful arm, and hematoma have a more direct anatomical relationship to venipuncture-associated vascular or tissue injury. Although these reactions are generally minor, their clinical relevance should not be judged solely by statistical significance. Arm pain, bruising, or localized swelling may cause discomfort or concern and adversely affect the donor's perception of the donation experience. These observations reinforce the practical importance of careful venipuncture, adequate compression of the donation site, and clear post-donation instructions.

Donor characteristics were associated with several whole-blood reaction categories. Painful arm was most frequent among donors aged 18-29 years (198/9,408 {2.10%}), whereas fatigue was most frequent among those aged 40-49 years (126/1,975 {6.38%}). Female whole-blood donors had markedly higher reported frequencies of fatigue (111/319 {34.80%} vs. 476/17,314 {2.75%}), bruising (88/319 {27.59%} vs. 302/17,314 {1.74%}), and painful arm (63/319 {19.75%} vs. 220/17,314 {1.27%}) than male donors. Physiological differences, such as lower average circulating and estimated blood volumes and differences in peripheral venous anatomy, may plausibly contribute to susceptibility to systemic or local reactions. However, these characteristics were not directly measured, female donors represented only 1.81% (319/17,633) of the whole-blood cohort, and the analyses were unadjusted. The magnitude of these sex-related differences should therefore be interpreted cautiously rather than as evidence of an independent effect of female sex. Previous evidence has similarly identified age, sex, estimated blood volume, and donation experience as factors associated with donor reactions [16].

Donation status was also associated with selected whole-blood reaction categories, although the absolute differences and corresponding effect sizes were generally small. Repeat donors had higher frequencies of bruising (129/4,987 {2.59%} vs. 261/12,646 {2.06%}) and fatigue (201/4,987 {4.03%} vs. 386/12,646 {3.05%}) than first-time donors. Conversely, vasovagal reactions were more frequent among first-time donors (48/12,646 {0.38%}) than repeat donors (5/4,987 {0.10%}). The small effect sizes for these associations illustrate the distinction between statistical and clinical significance in a large cohort. The greater frequency of vasovagal reactions among first-time donors is consistent with previous evidence identifying donation inexperience and donor-related physiological characteristics as susceptibility factors [16]. France et al. demonstrated that preventive measures, such as adequate hydration and the use of applied muscle tension, can reduce presyncopal reactions, particularly among susceptible donors [17].

The observed reaction profile also has practical implications for donor counseling. Before leaving the blood center, donors should receive clear information regarding commonly encountered post-donation symptoms, including fatigue, bruising, arm discomfort, and localized swelling, together with guidance regarding their expected course. Practical advice may include maintaining adequate hydration, gradually resuming normal activity, avoiding strenuous use of the donation arm immediately after donation, and maintaining appropriate compression of the venipuncture site. Donors should also be advised to contact the blood center if they develop persistent bleeding, progressive swelling, increasing arm pain, sensory symptoms, or prolonged or concerning weakness. Such counseling is relevant because even medically minor local complications may influence the donor's perception of the donation experience; Ranasinghe and Harrison reported that bruising could affect subsequent donor behavior [18]. Gupta and Bajpai likewise demonstrated that immediate and delayed adverse events may influence donors' intention to donate again [19]. These observations support individualized donor education and appropriate post-donation advice while recognizing that subsequent donor return and retention were not directly measured in the present study.

Plateletpheresis demonstrated a distinct delayed reaction profile, with hematoma (11/290 {3.79%}) and bruising (9/290 {3.10%}) being the most frequently reported reactions. Compared with whole-blood donors, plateletpheresis donors had higher unadjusted odds of hematoma (11/290 {3.79%} vs. 102/17,633 {0.58%}; OR: 6.77, 95% CI: 3.57-12.84), allergy (3/290 {1.03%} vs. 13/17,633 {0.07%}; OR: 14.12, 95% CI: 4.03-49.47), delayed bleeding (2/290 {0.69%} vs. 7/17,633 {0.04%}; OR: 17.37, 95% CI: 3.59-84.09}), and nerve irritation (2/290 {0.69%} vs. 9/17,633 {0.05%}; OR: 13.58, 95% CI: 2.91-63.35). The predominance of local venous-access events is biologically plausible because plateletpheresis involves prolonged venous cannulation, extracorporeal circulation, repeated component separation and return, and sustained venous access. Population-based and large automated-collection studies have similarly demonstrated procedure-specific patterns of donor complications [20,21]. Indian experience with apheresis procedures has also documented procedure-related adverse events and supports careful donor monitoring during component collection [22]. Nevertheless, the very small absolute numbers underlying several of the delayed outcomes in the present study, along with their wide confidence intervals, require cautious interpretation.

Citrate anticoagulation represents another important procedural difference between plateletpheresis and whole-blood donation. Citrate can produce transient reductions in ionized calcium and associated neuromuscular manifestations during apheresis [23]. Das et al. also demonstrated alterations in calcium and magnesium levels during automated plateletpheresis in healthy donors, further supporting the physiological basis of citrate-related intraprocedural symptoms [24]. Such citrate-associated manifestations are primarily acute or intraprocedural, whereas the present study focused on delayed symptoms reported after donors had left the blood center. Therefore, the observed delayed hematoma and bruising are more plausibly related to venous access than to citrate exposure. Similarly, delayed numbness or tingling categorized as nerve irritation should not be equated with intraprocedural citrate-associated paresthesia. Previous studies have reported that the frequency and pattern of donor reactions vary according to the collection procedure, supporting the need to consider donation modality when interpreting post-donation complaints [25-27].

Plateletpheresis subgroup analyses warrant particular caution. Following exact testing appropriate for sparse data, bruising was more frequent among female donors (3/17 {17.65%} vs. 6/273 {2.20%}) and repeat donors (2/11 {18.18%} vs. 7/279 {2.51%}), while fatigue was more frequent among female donors (3/17 {17.65%} vs. 2/273 {0.73%}). In contrast, hematoma was not significantly associated with age, sex, or donation status on exact testing. These subgroup findings should be regarded as exploratory because the plateletpheresis cohort comprised only 290 donors, including 17 females and 11 repeat donors. Consequently, apparently large percentage differences may arise from only a few events and should not be interpreted as evidence of independent risk factors. Yuan et al. identified sex, estimated blood volume, body size, and procedural characteristics as potential determinants of reactions during multicomponent apheresis; however, larger plateletpheresis cohorts with participant-level multivariable adjustment are required to determine whether the associations observed in the present study persist after accounting for potential confounding [28].

From a clinical perspective, the findings emphasize the importance of considering absolute frequency alongside relative measures of association. Large crude odds ratios for some plateletpheresis reactions arose from uncommon events; allergy occurred in 3/290 (1.03%), delayed bleeding in 2/290 (0.69%), and nerve irritation in 2/290 (0.69%) plateletpheresis donors. Conversely, more frequently reported symptoms such as fatigue and bruising may be relevant to donor comfort even when between-modality differences are not statistically significant. Moreover, the whole-blood and plateletpheresis groups differed in age, sex, and donation-status distributions, and the available analyses were unadjusted. Accordingly, the observed differences should be interpreted as associations rather than effects attributable solely to donation modality. This distinction is particularly important when considering procedure-specific counseling or preventive strategies.

Strengths, limitations, and future directions

This study has several strengths, including its prospective observational design, large overall donor cohort, inclusion of whole-blood and plateletpheresis donors from the same single tertiary-care blood center, and use of a structured four-week telephone follow-up approach. Follow-up was completed by 16,292/17,923 (90.9%) enrolled donors, allowing assessment of symptoms occurring outside the immediate post-donation observation period.

Several limitations should also be acknowledged. First, the single-center setting may limit generalizability to centers with different donor populations, collection procedures, or hemovigilance practices. Second, delayed reactions were based on donor self-report during telephone follow-up rather than contemporaneous clinical examination, creating potential for recall and symptom-classification bias. The four-week interval may have further increased recall uncertainty, particularly for mild or non-specific complaints such as fatigue. Pre-donation and intraprocedural reactions were outside the scope of the delayed off-site outcomes evaluated in this study. Third, 1,631/17,923 (9.1%) enrolled donors did not complete telephone follow-up. Donation-type-specific follow-up completion counts and characteristics of respondents vs. non-respondents were unavailable; therefore, potential differential non-response bias could not be assessed. Reaction-specific percentages were calculated using the enrolled donation-group denominators of 17,633 whole-blood and 290 plateletpheresis donors rather than respondent-only denominators and should be interpreted accordingly. The available aggregate records also do not permit reliable reconstruction of overlap between individual reaction categories. Fourth, the plateletpheresis cohort was substantially smaller than the whole-blood cohort, particularly among female (n=17) and repeat (n=11) donors, resulting in sparse cell counts and imprecise subgroup estimates. Fifth, only unadjusted analyses could be performed on the available aggregate data; consequently, potential confounding by age, sex, donation status, estimated blood volume, venous characteristics, and procedural factors could not be controlled for. Standardized ISBT/IHN severity grading was not prospectively available, limiting assessment of the clinical severity of individual delayed reactions. Finally, subsequent donation behavior was not evaluated; therefore, the present study cannot determine whether delayed reactions or post-donation follow-up affected donor return or long-term retention.

Future multicenter studies should include larger and more diverse plateletpheresis populations, participant-level data permitting multivariable adjustment, standardized hemovigilance definitions and severity grading, and shorter or serial follow-up intervals where feasible. Evaluation of subsequent donation behavior would also help determine whether particular delayed reactions influence donor return. Electronic follow-up systems may facilitate standardized surveillance and timely identification of post-donation complaints [29].

## Conclusions

Delayed adverse reactions following whole-blood donation and plateletpheresis showed distinct clinical patterns in this prospective single-center cohort. Among whole-blood donors, fatigue (587/17,633 {3.33%}), bruising (390/17,633 {2.21%}), and painful arm (283/17,633 {1.61%}) were the most frequently reported delayed reactions, whereas hematoma (11/290 {3.79%}) and bruising (9/290 {3.10%}) predominated among plateletpheresis donors. Plateletpheresis was associated with higher unadjusted odds of hematoma, allergy, delayed bleeding, and nerve irritation; however, several of these outcomes occurred in fewer than 1% of plateletpheresis donors, and the findings should be interpreted in the context of small event numbers and unadjusted comparisons. Donor age, sex, and donation status were associated with selected reaction categories, although findings for the plateletpheresis subgroup were exploratory due to the small numbers of female and repeat donors. These findings highlight the importance of recognizing delayed post-donation symptoms, providing donation-specific counseling, and interpreting relative associations alongside absolute frequencies. Larger multicenter studies with standardized severity assessment and multivariable analysis are required to confirm independent predictors of delayed donor adverse reactions.

## Appendices

Appendix 1

**Table 6:** STROBE checklist for observational studies. KGMU: King George's Medical University; STROBE: Strengthening the Reporting of Observational Studies in Epidemiology

Item	STROBE recommendation	Where addressed in the revised manuscript
1a. Title and abstract	Indicate the study design with a commonly used term in the title or abstract	Title and Abstract: study identified as a prospective observational study
1b. Abstract	Provide an informative and balanced summary of what was done and found	Structured Abstract: background, objective, methods, results, and conclusions
2. Background/rationale	Explain the scientific background and rationale for the investigation	Introduction: background on donor adverse reactions, delayed/off-site reactions, hemovigilance, and limitations of routine on-site surveillance
3. Objectives	State specific objectives, including prespecified hypotheses where applicable	Final paragraph of Introduction: primary objective and secondary objectives explicitly stated
4. Study design	Present key elements of study design early in the paper	Materials and Methods, “study design, setting, and reporting”: prospective observational design
5. Setting	Describe setting, location, and relevant dates, including recruitment and follow-up periods	Materials and Methods: single tertiary-care blood center, Department of Transfusion Medicine, KGMU, Lucknow; June 1, 2020, to May 31, 2021; four-week post-donation follow-up
6a. Participants	Give eligibility criteria and methods of selection of participants	Materials and Methods, “study population and donor eligibility”; key criteria in Table 1 and detailed criteria in appendix 1
6b. Matching	For matched studies, give matching criteria and numbers	Not applicable; this was not a matched study
7. Variables	Clearly define outcomes, exposures, predictors, potential confounders, and diagnostic criteria	Materials and Methods, “scope and classification of adverse reactions,” “operational assessment of delayed reactions,” and “outcome measures”
8. Data sources/measurement	Describe sources of data and methods of assessment for each variable	Materials and Methods: donor records and structured four-week telephone interview; operational symptom descriptions provided
9. Bias	Describe efforts to address potential sources of bias	Materials and Methods and limitations: structured interview approach described; potential recall bias, self-report/misclassification, and non-response bias explicitly acknowledged
10. Study size	Explain how the study size was determined	Materials and Methods, “sample size”: minimum n=920; all eligible consenting donors during the study period included; final N=17,923
11. Quantitative variables	Explain how quantitative variables were handled and, where applicable, why groups were chosen	Age analyzed using prespecified age categories; categorical donor characteristics summarized as n (%)
12a. Statistical methods	Describe statistical methods, including those used to control for confounding	Statistical analysis: Pearson's chi-square/exact testing as appropriate, crude ORs with 95% CIs, χ², df, and effect sizes. Analyses are explicitly identified as unadjusted
12b. Subgroups/interactions	Describe methods used to examine subgroups and interactions	Statistical analysis: subgroup analyses according to age, sex, and donation status
12c. Missing data	Explain how missing data were addressed	Follow-up completeness subsection: 16,292/17,923 (90.9%) completed follow-up; 1,631/17,923 (9.1%) did not. No retrospective imputation was performed
12d. Loss to follow-up	Explain how loss to follow-up was addressed	Follow-up completeness and participant-flow reporting; potential non-response bias acknowledged
12e. Sensitivity analyses	Describe any sensitivity analyses	Not performed; unavailable aggregate data did not support participant-level sensitivity analyses
13a. Participants	Report numbers at each stage of the study	Results and participant flow diagram: eligible/enrolled N=17,923; follow-up completed n=16,292 (90.9%); follow-up not completed n=1,631 (9.1%)
13b. Non-participation	Give reasons for non-participation at each stage	Available archived records identify incomplete follow-up but do not retain detailed reasons or donation-type-specific non-response; stated explicitly
13c. Flow diagram	Consider use of a flow diagram	Participant flow diagram included in the revised manuscript
14a. Descriptive data	Give participant characteristics and relevant exposures/confounders	Table 2: age, sex, donation status, and donation type
14b. Missing data for variables	Indicate number of participants with missing data for variables of interest	Overall follow-up completeness reported; donation-type-specific respondent counts cannot be reconstructed from retained aggregate records
14c. Follow-up time	Summarize follow-up time where appropriate	Four-week post-donation telephone follow-up specified
15. Outcome data	Report numbers of outcome events or summary measures	Results and Table 3: reaction-specific n (%) reported for whole-blood and plateletpheresis groups
16a. Main results	Give unadjusted estimates and, if applicable, confounder-adjusted estimates with precision	Table 3: crude ORs with 95% CIs; analyses clearly identified as unadjusted
16b. Category boundaries	Report category boundaries when continuous variables are categorized	Age-category boundaries reported in Table 2 and subgroup analyses
16c. Relative risk translation	Consider translating relative-risk estimates into meaningful absolute risk where relevant	Reaction frequencies n (%) are reported alongside crude ORs to provide absolute clinical context
17. Other analyses	Report subgroup and other analyses	Whole-blood and plateletpheresis subgroup analyses according to age, sex, and donation status
18. Key results	Summarize key results with reference to study objectives	Discussion, opening paragraphs
19. Limitations	Discuss limitations, including direction and magnitude of potential bias	Strengths, limitations, and future directions: single-center design, self-report, four-week recall interval, non-response, small plateletpheresis subgroups, lack of adjusted modeling and severity grading
20. Interpretation	Give cautious overall interpretation considering objectives, limitations, and other evidence	Discussion: associations interpreted as observational and exploratory; causal language avoided
21. Generalizability	Discuss external validity/generalizability	Limitations: single-center nature and need for multicenter validation explicitly stated
22. Funding	Give source of funding and role of funders	Funding/financial disclosure section of manuscript

Appendix 2

**Table 7:** Structured post-donation telephone interview schedule used for four-week donor follow-up.

Sections	Item/assessment	Operational description/question	Response/information recorded
A. Donor identification and follow-up	Donor identification/study number	Study identification number	-
	Donation type	Type of donation performed	☐ Whole-blood donation ☐ plateletpheresis
	Donation date	Date on which donation was performed	-
	Date of telephone follow-up	Date of post-donation telephone interview	-
	Follow-up status	Whether telephonic follow-up was completed	☐ Yes ☐ no
B. General post-donation assessment	General well-being	How was your general well-being following blood donation?	☐ No problem reported ☐ problem/discomfort reported
	Donation-related discomfort	Did you experience any discomfort during or after blood donation?	☐ No ☐ yes
	Off-site problem	Did you experience any problem after leaving the blood center?	☐ No ☐ yes
	Description of problem	If yes, what problem(s) did you experience?	Record according to the predefined categories below
C. Predefined delayed adverse reactions	Bruising	Bluish discoloration around the venipuncture site without swelling	☐ No ☐ yes
	Hematoma	Bluish discoloration associated with swelling around the venipuncture area	☐ No ☐ yes
	Painful arm	Pain or discomfort involving the phlebotomy arm following donation	☐ No ☐ yes
	Fatigue/weakness	Self-reported fatigue or generalized weakness following donation	☐ No ☐ yes
	Vasovagal reaction	Symptoms compatible with a delayed vasovagal episode reported after leaving the blood center	☐ No ☐ yes
	Allergic manifestations	Redness and/or itching reported following donation	☐ No ☐ yes
	Delayed bleeding	Bleeding from the venipuncture site occurring after leaving the blood center	☐ No ☐ yes
	Nerve irritation	Numbness or tingling involving the donation arm reported after leaving the blood center	☐ No ☐ yes
	Other post-donation complaint	Any additional problem reported by the donor	☐ No ☐ yes; specify:
D. Plateletpheresis-specific assessment	Intraprocedural citrate-associated symptoms	Tingling, burning sensation, muscle cramps, tetany, or related neuromuscular symptoms occurring during plateletpheresis	Classified as an acute/intraprocedural citrate-associated manifestation and not as a delayed off-site nerve irritation event
	Post-donation sensory symptoms	Numbness or tingling reported after the donor had left the blood center	Recorded under the delayed nerve irritation category according to the study classification
E. Further assessment/advice	Persistent/concerning complaint	Whether a persistent or clinically concerning complaint was reported	☐ No ☐ yes
	Further assessment	Whether the donor was advised to attend/contact the blood center for further assessment when required	☐ No ☐ yes
	Additional remarks	Any additional information relevant to the reported post-donation reaction	-

Appendix 3

**Table 8:** Detailed temporary and permanent donor eligibility and deferral criteria applied during the study period. *Donor eligibility was determined according to the applicable donor-selection criteria followed at the blood center during the study period, including the relevant eligibility criteria for donors receiving stable oral antidiabetic treatment. NSAID: nonsteroidal anti-inflammatory drug

Donor category	Criterion/condition	Eligibility requirement	Exclusion/deferral criterion or period
Whole-blood donation: temporary deferral criteria	Abortion	Completion of prescribed deferral interval	6 months
	Previous blood transfusion	Completion of prescribed deferral interval	12 months
	Major surgery	Completion of prescribed deferral interval	12 months
	Minor surgery	Completion of prescribed deferral interval	6 months
	Typhoid fever	Clinically recovered and prescribed interval completed	12 months after recovery
	Dengue/chikungunya	Full clinical recovery and prescribed interval completed	6 months after full recovery
	Malaria	Full clinical recovery and prescribed interval completed	3 months after full recovery
	Cold, influenza, cough, sore throat, or acute sinusitis	Symptoms resolved and donor afebrile	Deferred while symptomatic/febrile
	Tattoo	Completion of prescribed deferral interval	6 months
	Breastfeeding	Completion of lactation	Duration of lactation
	Non-live vaccines/toxoids	Completion of applicable interval	Cholera, typhoid, diphtheria, tetanus, injectable polio, and plague: 14 days
	Live attenuated vaccines	Completion of applicable interval	Oral polio, measles, mumps, yellow fever, Japanese encephalitis, typhoid, and hepatitis A: 28 days
	Swine-flu vaccination	Completion of prescribed interval	15 days
	Anti-rabies vaccination	Completion of prescribed interval	1 year
	Hepatitis B immunoglobulin	Completion of prescribed interval	1 year
	Other immunoglobulins	Completion of prescribed interval	1 year
	Hepatitis in family/close contact	Completion of prescribed interval	12 months
	Hepatitis immune globulin	Completion of prescribed interval	12 months
	Tuberculosis	Confirmed cure and prescribed interval completed	2 years after confirmation of cure
	Salicylates/aspirin and other NSAIDs	Completion of prescribed medication-free interval	3 days
	Ketoconazole/anthelmintic drugs, including mebendazole	Completion of prescribed medication-free interval	7 days after last dose
	Antibiotics	Completion of prescribed medication-free interval	2 weeks after last dose
	Oral antidiabetic treatment	Eligibility according to applicable stability requirements	Deferred when applicable eligibility requirements were not fulfilled
	Etretinate, acitretin, or isotretinoin	Completion of prescribed medication-free interval	1 month after last dose
	Radioactive contrast material	Completion of prescribed interval	8 weeks
	Medication of unknown nature	Medication identified and eligibility established	Deferred until medication details and eligibility could be established
Whole-blood donation: permanent medical deferral criteria	Malignancy	-	Cancer
	Cardiovascular disease	-	Heart disease
	Bleeding disorder	-	Abnormal bleeding tendencies
	Unexplained weight loss	-	Unexplained weight loss
	Diabetes/metabolic disease	-	Diabetes according to the applicable donor-selection criteria*
	Viral infection	-	Hepatitis B
	Renal disease	-	Chronic nephritis
	HIV/AIDS-related condition	-	Signs and symptoms suggestive of AIDS
	Hematological disease	-	Polycythemia vera
	Respiratory disease	-	Asthma
	Neurological disease	-	Epilepsy
	Chronic infection	-	Leprosy
	Psychiatric disease	-	Schizophrenia
	Endocrine disease	-	Endocrine disorders
	Insulin therapy	-	Insulin treatment
Plateletpheresis-specific criteria	Age	18-60 years	<18 or >60 years
	Body weight	≥50 kg	<50 kg
	General medical fitness	Medically fit for plateletpheresis	Medically unfit
	ABO/Rh compatibility	Same ABO and Rh compatibility as the intended recipient/patient, where applicable	Required compatibility not fulfilled
	Hemoglobin	≥12.5 g/dL	<12.5 g/dL
	Platelet count	≥150×10⁹/L	<150×10⁹/L
	Transfusion-transmitted infection screening	Nonreactive for HIV-1, HIV-2, hepatitis B virus, hepatitis C virus, syphilis, and malaria	Reactive for any mandatory transfusion-transmitted infection
	NSAID/aspirin exposure	No NSAID/aspirin use during the preceding 72 h	NSAID/aspirin use during the preceding 72 h
	Venous access	Suitable antecubital veins for phlebotomy	Unsuitable antecubital venous access
	Interval between plateletpheresis procedures	≥48 h; no more than two procedures/week or 24 procedures/year	<48 h or frequency exceeding two procedures/week or 24 procedures/year
	Previous plateletpheresis reaction	No history of donor reaction during previous plateletpheresis donation	History of donor reaction during previous plateletpheresis donation
	Other medication/deferral conditions	Meets applicable medication and donor-deferral criteria	Presence of an applicable medication-related or other deferral condition
